# The effects and mechanism of paeoniflorin in promoting osteogenic differentiation of MC3T3-E1

**DOI:** 10.1186/s13018-022-02965-1

**Published:** 2022-02-14

**Authors:** Wei Guo, Xiao-guang Yang, Yu-lin Shi, Hong Wang

**Affiliations:** Department of Rehabilitation Medicine, Xiangyang Central Hospital, Affiliated Hospital of Hubei University of Arts and Science, No. 136, Jingzhou Road, Xiangyang, 441021 Hubei China

**Keywords:** Paeoniflorin, Wnt/β-catenin pathways, MC3T3-E1 cells, Osteogenic differentiation, Osteoclastogenesis

## Abstract

**Background:**

The incidence of osteoporosis and osteoporotic fractures is increasing every year. Traditional Chinese Medicine (TCM) can shed new light on the treatment of osteoporosis. This study aimed to explore the role and mechanism of paeoniflorin in promoting osteogenic differentiation of an osteoblast precursor cell line (MC3T3-E1).

**Methods:**

MC3T3-E1 cells were cultured in osteogenic induction medium (OIM) and OIM combined with different concentrations of paeoniflorin. The optimal dose of paeoniflorin was assessed by a cell counting kit-8 (CCK-8) assay. Then, alkaline phosphatase (ALP) and Alizarin Red S (ARS) staining were performed to assess the osteogenic capacity of paeoniflorin. The transcription of osteogenic genes and the expression of osteogenic proteins were assessed by RT-PCR and Western blotting, respectively. The transcription of Wnt/β-catenin signaling pathway genes and proteins was assessed by RT-PCR and Western blotting, respectively. Finally, Dickkopf-1 (DKK-1), a Wnt/β-catenin signaling pathway inhibitor, was used to identify whether the Wnt/β-catenin signaling pathway was involved in the osteogenic differentiation of paeoniflorin. Osteoclastogenesis in RAW264.7 cells was identified by tartrate-resistant acid phosphatase (TRAP) staining.

**Results:**

At concentrations ranging from 0.1 to 100 μM, paeoniflorin was not cytotoxic to MC3T3-E1 cells. Paeoniflorin significantly increased the osteogenic differentiation of MC3T3-E1 cells in a dose-dependent manner. Moreover, paeoniflorin significantly increased osteogenic differentiation gene and protein expression. Through bioinformatic analysis, paeoniflorin-affected genes were found to be involved in different signaling pathways, such as the Wnt/β-catenin signaling pathway. Paeoniflorin enhanced β-catenin and CyclinD1 expression compared with that of the control groups. DKK-1 partially reversed the promoting effects of paeoniflorin in promoting osteogenic differentiation of MC3T3-E1 cells. Moreover, paeoniflorin inhibited the osteoclastogenesis of RAW264.7 cells.

**Conclusion:**

Paeoniflorin promotes osteogenic differentiation in MC3T3-E1 cells by regulating the Wnt/β-catenin pathway. Paeoniflorin is a potential therapeutic agent for the treatment of osteoporosis.

## Background

Osteoporosis is a metabolic and systemic skeletal system disease [1]. The pathophysiological characteristics of osteoporosis include decreased bone mass, increased bone fragility, and bone microstructure destruction [2, 3]. The main population affected by osteoporosis is menopausal women [4, 5]. According to European epidemiological statistics in 2010, among people aged 50–84, approximately 6% of men and 21% of women have been diagnosed with osteoporosis [6]. With the increasing aging population, the number of patients with osteoporosis is also increasing every year [7, 8].

The prevention and treatment of osteoporosis includes many aspects, such as appropriate exercise, adequate sunshine, and cessation of smoking and alcohol consumption [9]. Anti-osteoporosis drugs included bisphosphonates, calcitonins, selective estrogen receptor modulators, parathyroid hormone analogs, strontium salts, active vitamin D and its analogs, and RANKL inhibitors [10–13]. However, these drugs have some disadvantages. Therefore, it is necessary to actively look for other treatment options for osteoporosis. Traditional Chinese Medicine provides new ideas for the treatment of osteoporosis.

Paeoniflorin is a traditional Chinese herbal medicine and is the main component of the total glucosides of paeony [14]. Traditional paeoniflorin exhibits anti-inflammatory and diuretic effects [15, 16]. In 1998, the US Food and Drug Administration (FDA) approved the sale of paeoniflorin [17]. Paeoniflorin has many pharmacological activities, such as anti-inflammatory activity, immune regulation, and antiallergic, analgesic, antioxidative, and antitumor activities [18–20]. A previous study found that paeoniflorin attenuates dexamethasone-induced apoptosis of osteoblast cells and promotes bone formation by regulating the AKT/mTOR/autophagy signaling pathway [21]. These results suggest that paeoniflorin stimulates osteoblastogenesis and can be used as an adjuvant natural medicine for bone diseases such as osteoporosis. Transcription factors, such as runt-related transcription factor 2 (RUNX2) and osterix (OSX), have an essential role in osteoblast differentiation and bone formation [22–24].

Osteogenic differentiation is mediated by the expression of RUNX2, which is considered as the master regulator that controls osteoblast differentiation, gene expression, and function [25].

Previous studies found osterix in c-Abl-/- calvarial osteoblasts was downregulated, indicating that the positive role of c-Abl in osteoblast differentiation could be mediated by osterix [26].

Several studies have shown that the Wnt/β-catenin signaling pathway is involved in the osteogenic differentiation of multiple stem cells and osteoblasts [27, 28]. In the WNT pathway, WNT binds to Frizzled and LRP, which transduce the signal to downstream components of the various branches of Wnt signal transduction [29]. Therefore, activating the Wnt/β-catenin signaling pathway is crucial for osteogenic differentiation and thus may be a target for osteoporosis. However, whether paeoniflorin stimulates osteogenic differentiation of MC3T3-E1 cells through the Wnt/β-catenin signaling pathway is unknown.

Accordingly, we determined the effects of paeoniflorin on the osteogenic differentiation of MC3T3-E1 cells.

## Material and methods

### Cell source and preparation

**Fig. 1:**
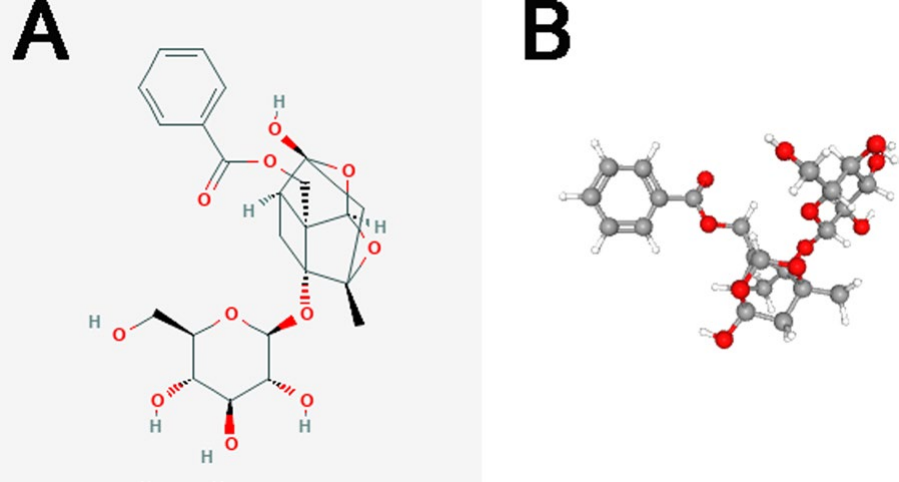
2D and 3D chemical structures of paeoniflorin

Preosteoblastic MC3T3-E1 cells were obtained from the American Type Culture Collection (ATCC) (Manassas, Virginia, USA). Paeoniflorin (purity, > 99%) was obtained from Sigma-Aldrich (St. Louis, MO, USA). The 2D and 3D chemical structures of paeoniflorin are shown in Fig. 1A, B, respectively. Paeoniflorin was dissolved in DMSO, with a maximum DMSO concentration of 0.1% used for the assays. The concentration of paeoniflorin ranged from 0.1 to 100 μM. Primary human osteoblasts (HOBs) obtained from normal human hip samples were purchased commercially (Procell, Wuhan, Wubei Province, China).

### Osteogenic induction

Briefly, osteogenic differentiation was induced primarily by β-glycerol phosphate (10 mM), dexamethasone (10^−8^ M), and ascorbic acid (50 µM). MC3T3-E1 cells were seeded at 3 × 10^5^/well in 6-well culture plates. When reaching approximately 80–90% confluence, the culture medium of the MC3T3-E1 and HOBs was changed to osteogenic differentiation medium. Culture media were changed every 2 days.

### Cell counting kit-8 (CCK-8) assay

Cell proliferation was analyzed using a CCK-8 assay kit following the manufacturers’ manual. In brief, MC3T3-E1 cells were divided into a control group and groups with different concentrations of paeoniflorin. After incubation of the samples at 37 °C for 1, 3, and 5 days, 10 μL CCK-8 solution was added to the MC3T3-E1 cells and incubated for another 2 h. A Thermomax Microplate Reader (Molecular Devices, Sunnyvale, CA, USA) was used to assess cell viability. The cell viability (%), normalized to the control group, was calculated.

### ALP staining and ALP activity

MC3T3-E1 cells or HOBs were seeded at a concentration of 1 × 10^5^ cells/well in 24-well culture plates and cultivated in an incubator for 24 h. After 7 days of culture, the osteogenic capacity of MC3T3-E1 cells or HOBs was analyzed by ALP staining. Briefly, MC3T3-E1 cells were washed with PBS three times and fixed with 4% paraformaldehyde for 15 min. Then, MC3T3-E1 cells were incubated with 5-bromo-4-chloro-3-indolyl phosphate (BCIP)/nitro blue tetrazolium (NBT) (Tiangen Biotech Co., Beijing, China) for 30 min. Then, the reaction was stopped by adding distilled water. Images were captured with a Nikon microscope (Nikon, Tokyo, Japan). In a pH 10.2 environment, nitrophenol phosphate was used as a substrate, and the absorbance at 405 nm was measured by a Thermomax Microplate Reader (Molecular Devices, Sunnyvale, CA, USA).

### Alizarin Red S staining

MC3T3-E1 cells were seeded at a concentration of 1 × 10^5^ cells/well in 24-well culture plates and cultivated in an incubator for 24 h. After 21 days of culture, mineralization was analyzed by Alizarin Red S staining. MC3T3-E1 cells or HOBs were divided into the following groups: the control group and groups with different concentrations of paeoniflorin. In brief, the culture plates were washed three times with sterile distilled water to remove nonadherent cells. MC3T3-E1 cells or HOBs were fixed with 4% paraformaldehyde for 15 min. Alizarin Red S staining was performed by incubation for 30 min with Alizarin Red S solution (2%, pH 4.4). Images were captured with a Nikon microscope (Nikon, Tokyo, Japan). For quantitative measurement, the alizarin red stain was dissolved in a 10% cetylpyridinium chloride monohydrate solution for 30 min. MC3T3-E1 cells or HOBs were incubated for 30 min. The optical density of the samples was measured in a plate reader at 540 nm.

### Real-time PCR

Messenger RNAs were extracted using the RNeasy Plus Mini Kit (Qiagen, 74,136). RNA concentration and purity were assessed by its 260/280 optical density (OD) ratio. Reverse transcription was performed with M-MLV-RT (Promega, Wallisellen, Switzerland) and random primers (Roche Diagnostics). The thermocycling conditions were 94 °C for 40 s, 60 °C for 40 s, and 72 °C for 30 s, followed by incubation at 4 °C indefinitely. The primer sequences were as follows; ALP: forward, 5ʹ-TGACCTTCTCTCCTCCATCC-3ʹ, reverse, 5ʹ-CTTCCTGGGAGTCTCATCCT-3ʹ; OCN: forward, 5ʹ-CTTGAAGACCGCCTACAAAC-3ʹ, reverse, 5ʹ-GCTGCTGTGACATCCATAC-3ʹ; OSX: forward, 5ʹ-TCCTGTAGATCCGAGCACCA-3ʹ, reverse, 5ʹ-CTGCTGCTGTTGTTGCTGTT-3ʹ; GAPDH: forward, 5ʹ-AGCCATGTACGTAGCCATCC-3ʹ, reverse, 5ʹ-CTCTCAGCAGTGGTGGTGAA-3ʹ. The β-actin gene was used as an internal control.

### Western blot

Cell lysates were prepared by a RIPA lysis solution. Then, the protein concentration was assessed by the Lowry method using a BCA Protein Assay Kit (P0011, Beyotime, Shanghai, China). After 30 min of polymerization, gel electrophoresis was used to separate the protein samples. After the bromophenol blue dye ran approximately 1 cm into the separating gel, the electrophoresis was stopped. The resolved proteins were transferred to PVDF membranes (Millipore, USA). Then, blocking solution was added and incubated for 1 h at room temperature. Primary antibodies were diluted in blocking solution and added to the PVDF membrane. Following rewarming, the membranes were washed with PBS 3 times for 10 min. The PVDF membrane was then incubated with HRP-conjugated goat anti-rabbit antibody (1:2000, Santa Cruz). Immunoreactive proteins were detected by using an ECL Kit (Beyotime Biotech, Shanghai, China), and the gray value of the protein bands was calculated by ImageJ software.

### Cell culture

RAW264.7 cells (a mouse leukemic monocyte macrophage cell line) were obtained from the ATCC. RAW264.7 cells were maintained in Dulbecco's modified Eagle's medium (DMEM) containing 10% fetal bovine serum (FBS) and supplemented with 1% penicillin, streptomycin, and amphotericin at 37 °C in a humidified 5% CO_2_ atmosphere. RAW264.7 cells (2 × 10^5^ cells/well in a 6-well plate) were pretreated with 0.1 to 100 μM paeoniflorin for 2 h and then stimulated with RANKL (100 ng/ml) for 7 days to induce osteoclastogenesis as previously described [30].

### TRAP staining

Following the seven-day induction of osteoclastogenesis, the cells were fixed using 4% paraformaldehyde for 10 min. After washing with PBS 3 times, the plates were incubated for 10 min in TRAP staining solution at 37 °C in the dark according to the instructions of the TRAP staining kit (Sigma-Aldrich, St. Louis, MO, USA). The cells were observed and photographed under a Nikon microscope (Nikon, Tokyo, Japan). TRAP-positive cells with three or more nuclei were counted.

### Statistical analysis

All results are expressed as the mean ± standard deviation (SD). One-way analysis of variance followed by Dunnett's post hoc test was performed to analyze significant differences between groups. The statistical significance was set as *P* < 0.05.

## Results

### CCK-8 assay

**Fig. 2:**
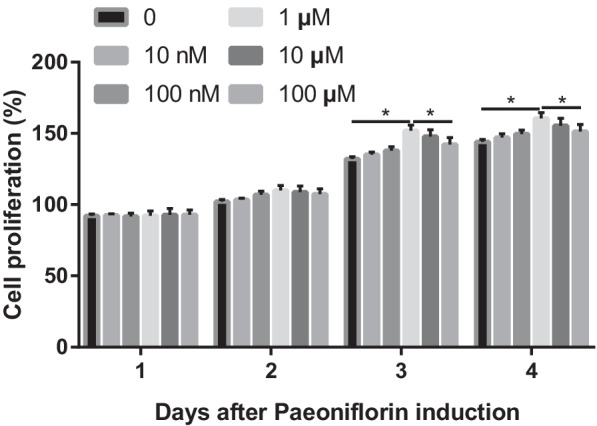
Cytotoxic effect of paeoniflorin on MC3T3-E1 cells. The experimental data are expressed as the mean ± standard deviation. Significance analysis of the experimental data for each group was performed using one-way analysis of variance and Tukey's multiple comparisons test. *P < 0.05

Different concentrations of paeoniflorin were used to stimulate MC3T3-E1 cells for 1–3 days. Concentrations of paeoniflorin between 0 and 100 μM did not cause cytotoxicity. However, concentrations above 10 μM significantly reduced cell viability in a dose-dependent manner. Compared with the control, the concentration of 1 μM had no significant effect on the cells (Fig. 2).

### ALP and ARS staining

**Fig. 3:**
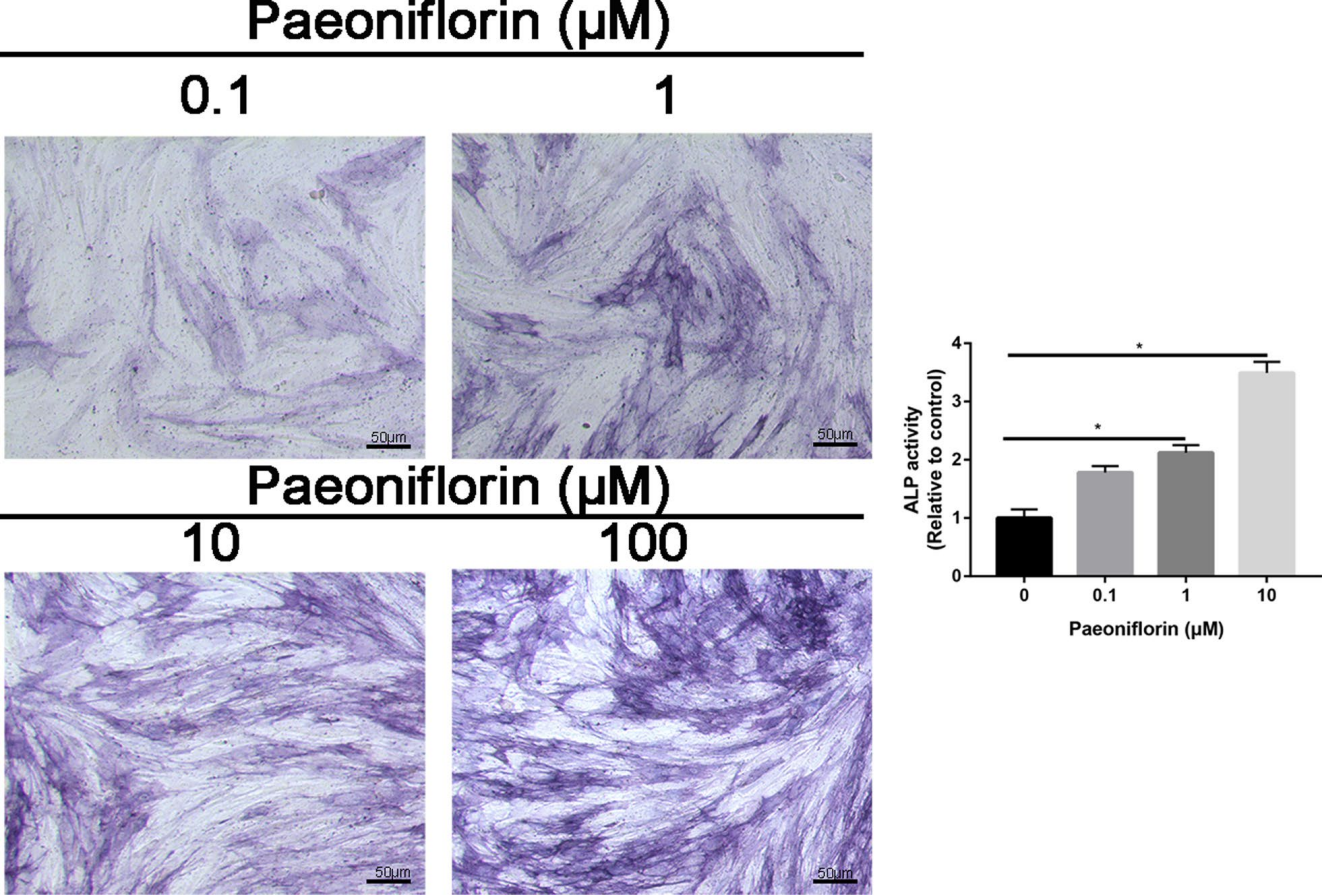
ALP staining of MC3T3-E1 cells treated with control or different concentrations of paeoniflorin

Differentiation was verified by ARS and ALP staining. The results from ALP staining showed that paeoniflorin treatment dose-dependently increased ALP activity. The most pronounced effect on these parameters was found in the 100 μM dose group (Fig. 3).

**Fig. 4:**
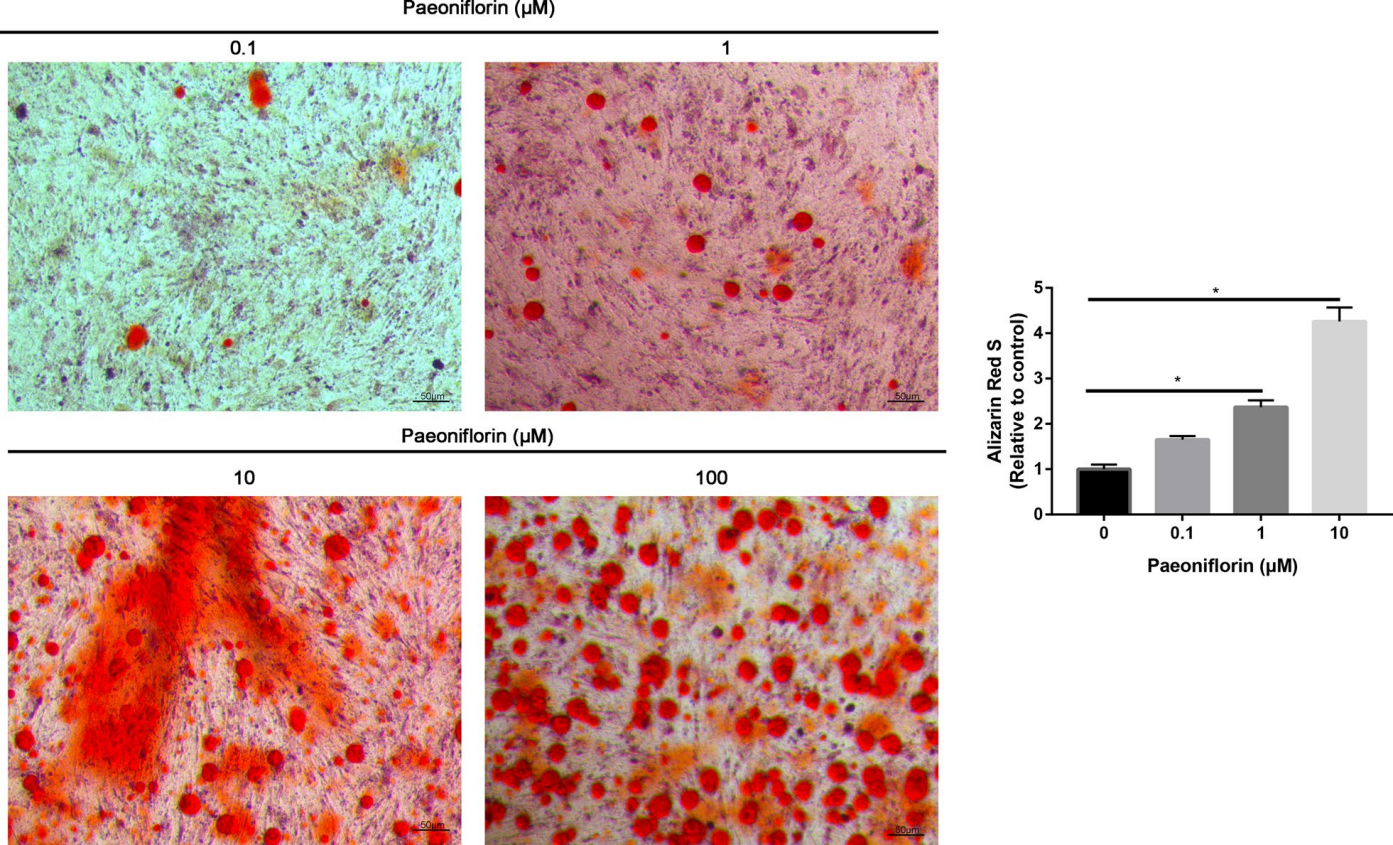
ARS staining and quantitative analysis of MC3T3-E1 cells treated with control or different concentrations of paeoniflorin

Calcium precipitation was measured by ARS staining to probe the efficiency of osteogenesis. ARS staining revealed that the paeoniflorin-treated MC3T3-E1 cells (100 μM) had nearly twofold more calcium deposition and mineralization nodules than did the control group (Fig. 4). The ARS quantification results were consistent with the ARS staining results. The ALP and ARS staining results of HOBs were consistent with those of the MC3T3-E1 cells, which indicated that paeoniflorin enhanced the osteogenic differentiation of HOBs.

### PCR assay

**Fig. 5:**
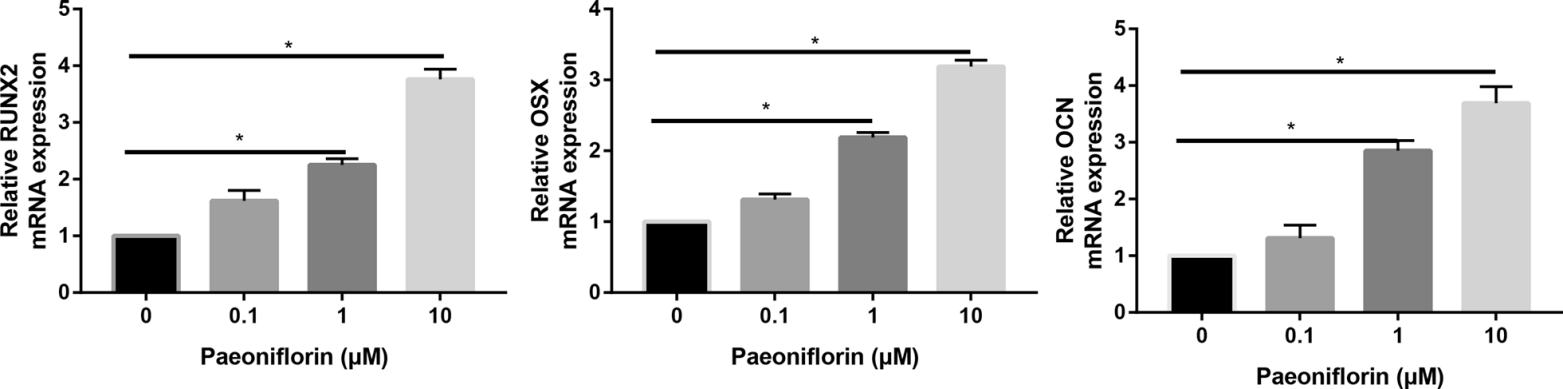
qRT-PCR analysis of the osteogenic genes RUNX2, OSX, and OCN in osteogenic-induced MC3T3-E1 cells

To further confirm the conclusion that paeoniflorin has an anabolic effect on bone, we investigated the effect of paeoniflorin on osteoblast marker expression. ALP, OCN, and OSX expression levels were significantly increased in paeoniflorin-treated MC3T3-E1 cells compared to that of the control group (*P* < 0.01). With paeoniflorin treatment at 50 μg/ml, the ALP, OCN, and OSX mRNA expression levels increased significantly to 4.56-, 3.89-, and 5.13-fold higher than that of the control group, respectively (Fig. 5).

### Western blot assay

**Fig. 6:**
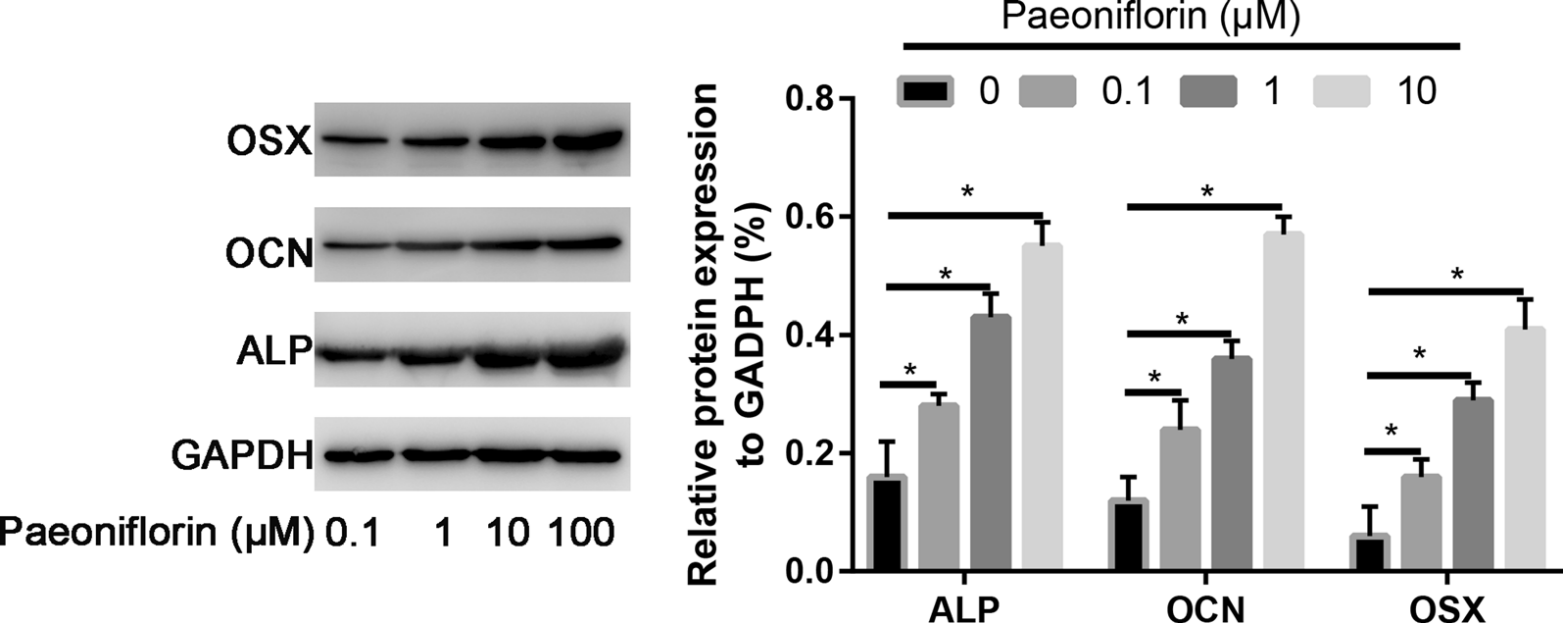
Western blot analysis of the osteogenic genes RUNX2, OSX, and OCN in osteogenic-induced Sat- and Mac-ASCs

As illustrated in Fig. 6, the ALP, OCN, and OSX protein expression levels increased 1.99-, 2.34-, and 2.15-fold, respectively, after paeoniflorin treatment compared to the control group (*P* < 0.05). In addition, 50 μg/ml paeoniflorin increased the ALP, OCN, and OSX expression levels more strongly (2.15-fold) than compared to the other concentrations of paeoniflorin, with statistically significant differences (*P* < 0.05, Fig. 6).

### Paeoniflorin activated the Wnt/β-catenin signaling pathway

**Fig. 7:**
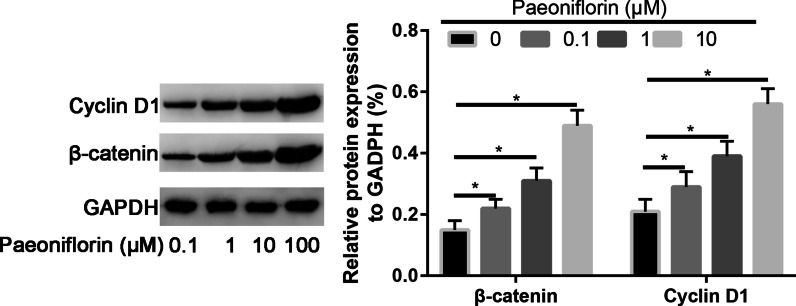
Western blot analysis of the osteogenic genes cyclin D1 and β-catenin in MC3T3-E1 cells

We then investigated the change in the active β-catenin and cyclin D1 levels in MC3T3-E1 cells by Western blotting. The Western blotting results showed the upregulation of active β-catenin and Cyclin D1 protein expression in MC3T3-E1 cells after paeoniflorin treatment. In addition, 50 μg/ml paeoniflorin increased the active β-catenin and cyclin D1 levels even more strongly than other concentrations of paeoniflorin (Fig. 7).

### Inhibition of the Wnt/β-catenin signaling pathway partially reversed the promoting effects of paeoniflorin on MC3T3-E1 cells

**Fig. 8:**
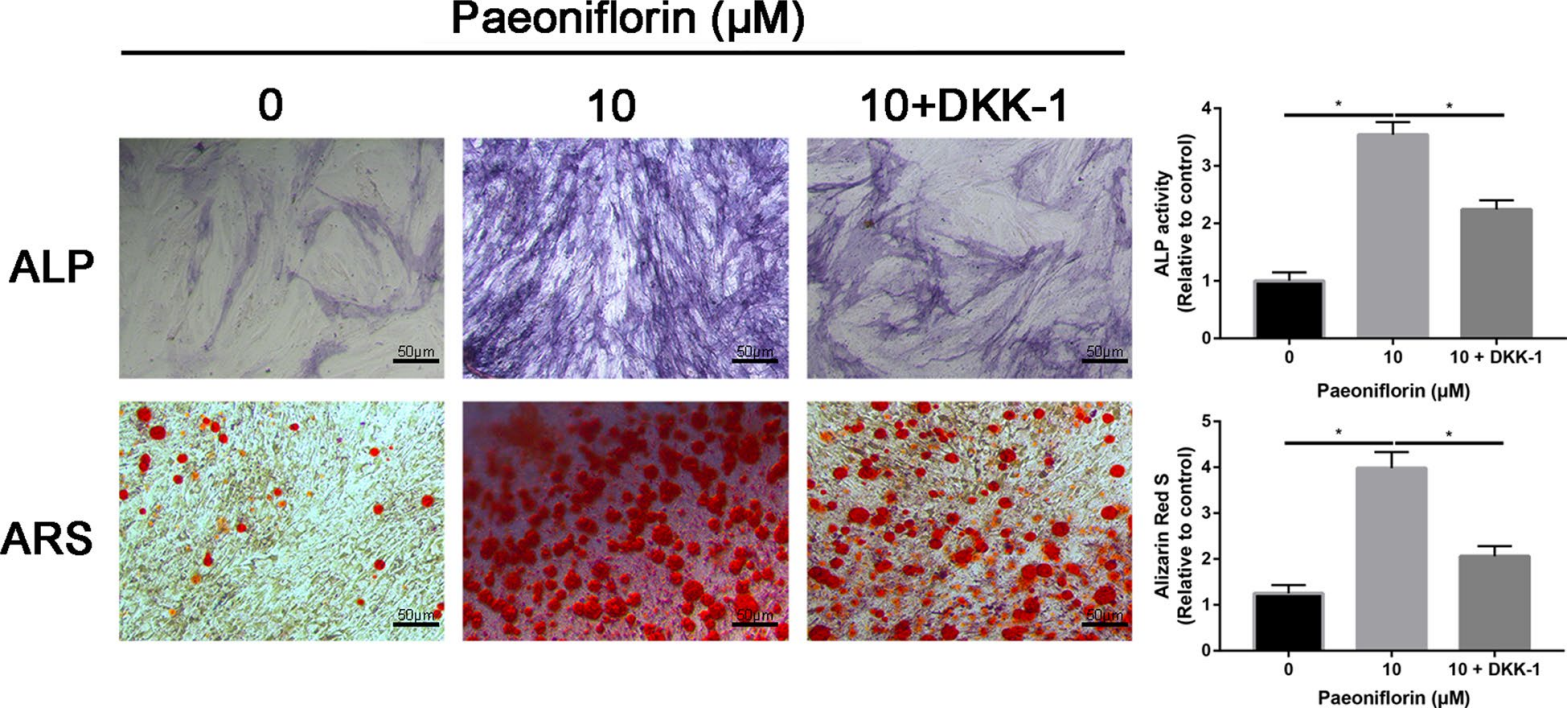
ALP and ARS staining of MC3T3-E1 cells in the control, paeoniflorin, and paeoniflorin + DKK-1 groups

To investigate the regulatory effect of the Wnt signaling pathway on paeoniflorin-induced osteoblast differentiation of MC3T3-E1 cells, the Wnt inhibitor Dickkopf-related protein 1 (DKK-1) was used. The results were consistent with previous findings, and paeoniflorin significantly enhanced the osteogenic differentiation of MC3T3-E1 cells, which was partially suppressed by the Wnt inhibitor DKK-1 (Fig. 8).

**Fig. 9:**
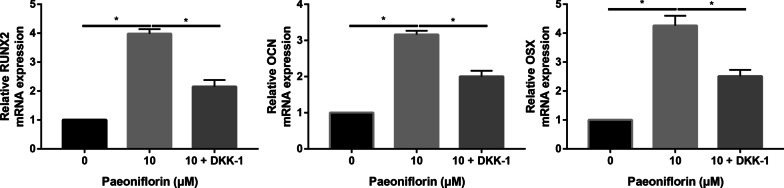
qRT-PCR analysis of the osteogenic genes RUNX2, OSX, and OCN in the control, paeoniflorin, and paeoniflorin + DKK-1 groups

**Fig. 10:**
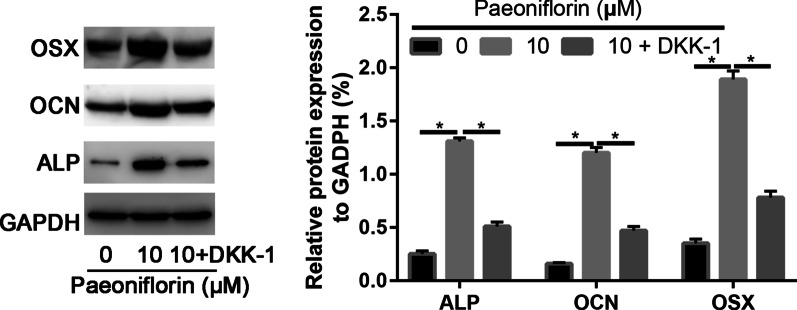
Western blot analysis of the osteogenic markers RUNX2, OSX, and OCN in the control, paeoniflorin and paeoniflorin + DKK-1 groups

Paeoniflorin significantly enhanced ALP, OCN, and OSX mRNA expression in MC3T3-E1 cells, which was partially suppressed by the Wnt inhibitor DKK-1 (Fig. 9). The ALP, OCN, and OSX expression tendencies were in accordance with the real-time PCR results (Fig. 10).

### Paeoniflorin inhibited the osteoclastogenesis of RAW264.7 cells

To determine the effect of paeoniflorin on RANKL-induced osteoclastogenesis, we pretreated RAW264.7 cells with different concentrations of paeoniflorin in the presence of RANKL and then evaluated the formation of osteoclasts.

**Fig. 11:**
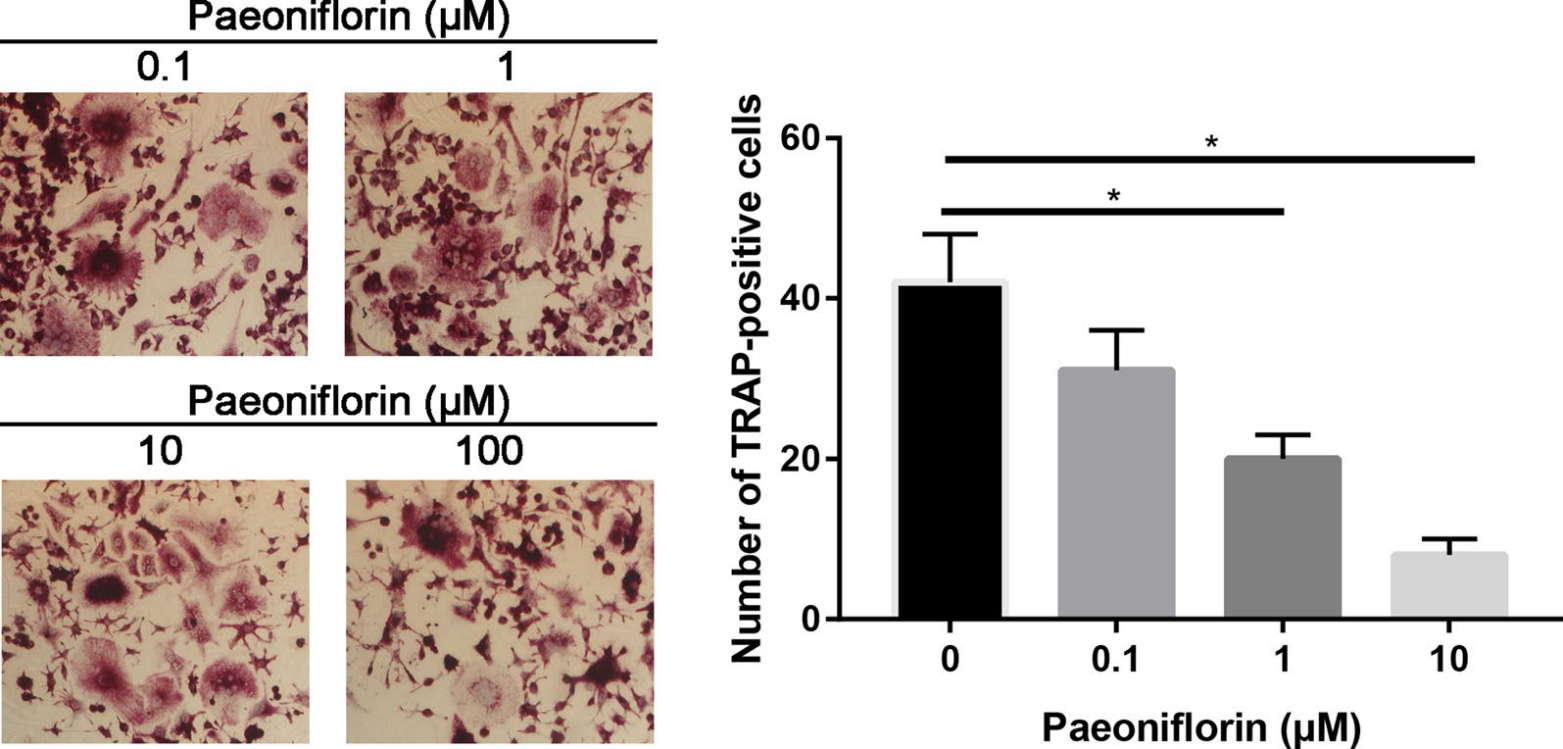
TRAP staining of different treatment groups

**Fig. 12:**
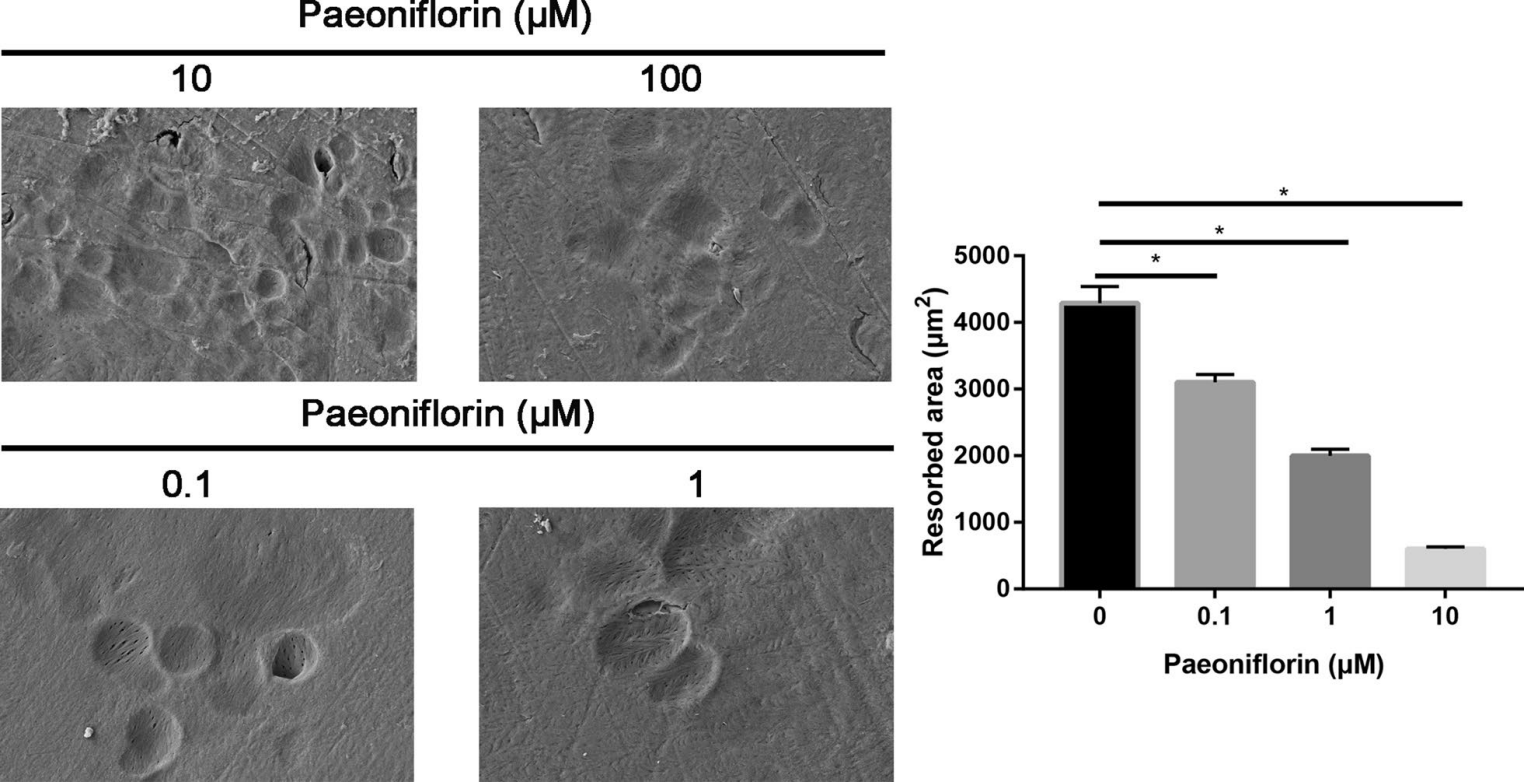
SEM of different treatment groups

RAW264.7 cells cultured in the presence of RANKL formed TRAP + cells. However, paeoniflorin treatment reduced the number of RANKL-induced osteoclasts in a dose-dependent manner (Fig. 11, 12). These data thus suggest that aconine inhibited RANKL-induced osteoclastogenesis.

## Discussion

This is the first study to explore the role and mechanism of paeoniflorin in promoting the osteogenic differentiation of MC3T3-E1 cells. We found that paeoniflorin significantly promoted osteogenic differentiation of MC3T3-E1 cells through the Wnt/β-catenin signaling pathway.

We first identified the optimal dose of paeoniflorin for MC3T3-E1 cell viability. In a certain concentration range of paeoniflorin, the results displayed a dose-dependent effect of paeoniflorin on the proliferation and osteogenic differentiation of MC3T3-E1 cells in vitro. These promoting effects were further verified by real-time PCR and Western blot assays. Yang et al. [21] revealed that paeoniflorin promotes bone formation by regulating the AKT/mTOR/autophagy signaling pathway in an animal model. Paeoniflorin has been reported previously to increase osteoblastogenesis in in vitro and in vivo models [31]. However, the influence of paeoniflorin on MC3T3-E1 cells remains rather unclear.

To explore the mechanism by which paeoniflorin promotes the osteogenic differentiation of MC3T3-E1 cells, the STITCH database was searched to identify the target genes of paeoniflorin. Further bioinformatic analysis revealed that these target genes were mainly enriched in the Wnt/β-catenin signaling pathway. Therefore, a further series of experiments were performed to identify the mechanism by which paeoniflorin promotes the osteogenic differentiation of MC3T3-E1 cells. Cyclin D1 and β-catenin expression were measured in control and paeoniflorin-treated MC3T3-E1 cells. Paeoniflorin significantly augmented cyclin D1 and β-catenin expression, which suggested that paeoniflorin enhanced the Wnt/β-catenin signaling pathway. The Wnt/β-catenin signaling pathway includes classic Wnt/β-catenin signaling pathways and the nonclassical Wnt/β-catenin signaling pathway [32, 33]. These two pathways are both involved in the functional regulation of osteogenic differentiation of osteoblasts [34, 35]. MC3T3-E1 cells are in a dormant stable state under normal conditions. MC3T3-E1 cells can differentiate into osteoblasts under osteogenic induction. Wnt ligands can activate β-catenin by binding to membrane receptors.

To further identify that the Wnt/β-catenin signaling pathway was involved in the osteogenic differentiation of MC3T3-E1 Cells, the Wnt/β-catenin signaling pathway inhibitor DKK-1 was used to block the pathway. We found that DKK-1 partially reversed paeoniflorin-mediated promotion of the osteogenic differentiation of MC3T3-E1 cells. Further real-time PCR and Western blot assays identified the ALP and ARS staining results. These results suggest to us that paeoniflorin stimulates osteogenic differentiation by regulating the Wnt/β-catenin signaling pathway.

There were limitations of this study. MC3T3 cells are a cell line and were used to assess cell differentiation and mineralization. This cell line is different from primary cells. The present results may be confirmed by using an additional cell line, such as mesenchymal stem cells. Moreover, this study focused on the role and mechanism of paeoniflorin through in vitro experiments. Future studies should be performed in vivo with animals to confirm our results. Further in-depth toxicity studies should be also performed in the future.

## Conclusion

In conclusion, our data demonstrated for the first time that paeoniflorin enhances the osteogenic differentiation potential of MC3T3-E1 cells by directly upregulating the Wnt/β-catenin signaling pathway. Moreover, paeoniflorin inhibited the osteoclastogenesis of RAW264.7 cells. Paeoniflorin may be a potential therapeutic target for osteoporosis.

## Data Availability

All the data will be available upon request from the corresponding author of this paper.

## Abbreviations

TCM: Traditional Chinese Medicine
OIM: Osteogenic induction medium
CCK-8: Cell counting kit-8
DKK-1: Dickkopf-1
FDA: Food and Drug Administration
BCIP: 5-Bromo-4-chloro-3-indolyl phosphate
NBT: Nitro blue tetrazolium
OD: Optical density
SD: Standard deviation
